# Inclusion of the glucocorticoid receptor in a hypothalamic pituitary adrenal axis model reveals bistability

**DOI:** 10.1186/1742-4682-4-8

**Published:** 2007-02-14

**Authors:** Shakti Gupta, Eric Aslakson, Brian M Gurbaxani, Suzanne D Vernon

**Affiliations:** 1 Division of Viral and Rickettsial Diseases, National Center for Zoonotic, Vector-Borne, and Enteric Diseases, Centers for Disease Control and Prevention, 600 Clifton Rd, MS-A15, Atlanta, Georgia 30333, USA

## Abstract

**Background:**

The body's primary stress management system is the hypothalamic pituitary adrenal (HPA) axis. The HPA axis responds to physical and mental challenge to maintain homeostasis in part by controlling the body's cortisol level. Dysregulation of the HPA axis is implicated in numerous stress-related diseases.

**Results:**

We developed a structured model of the HPA axis that includes the glucocorticoid receptor (GR). This model incorporates nonlinear kinetics of pituitary GR synthesis. The nonlinear effect arises from the fact that GR homodimerizes after cortisol activation and induces its own synthesis in the pituitary. This homodimerization makes possible two stable steady states (low and high) and one unstable state of cortisol production resulting in bistability of the HPA axis. In this model, low GR concentration represents the normal steady state, and high GR concentration represents a dysregulated steady state. A short stress in the normal steady state produces a small perturbation in the GR concentration that quickly returns to normal levels. Long, repeated stress produces persistent and high GR concentration that does not return to baseline forcing the HPA axis to an alternate steady state. One consequence of increased steady state GR is reduced steady state cortisol, which has been observed in some stress related disorders such as Chronic Fatigue Syndrome (CFS).

**Conclusion:**

Inclusion of pituitary GR expression resulted in a biologically plausible model of HPA axis bistability and hypocortisolism. High GR concentration enhanced cortisol negative feedback on the hypothalamus and forced the HPA axis into an alternative, low cortisol state. This model can be used to explore mechanisms underlying disorders of the HPA axis.

## Background

The hypothalamic pituitary adrenal (HPA) axis represents a self-regulated dynamic feedback neuroendocrine system that is essential for maintaining body homeostasis in response to various stresses. Stress can be physical (e.g. infection, thermal exposure, dehydration) and psychological (e.g. fear, anticipation). Both physical and psychological stressors activate the hypothalamus to release corticotropin releasing hormone (CRH). The CRH is released into the closed hypophyseal portal circulation, stimulating the pituitary to secrete adrenocorticotropic hormone (ACTH). ACTH is released into the blood where it travels to the adrenals, inducing the synthesis and secretion of cortisol from the adrenal cortex. Cortisol has a negative feedback effect on the hypothalamus and pituitary that further dampens CRH and ACTH secretion [1].

Cortisol affects a number of cellular and physiological functions to maintain body homeostasis and health. Cortisol suppresses inflammation and certain immune reactions, inhibits the secretion of several hormones and neuropeptides and induces lymphocyte apoptosis [1,2]. These widespread and potent effects of cortisol demand that the feed forward and feedback loops of the HPA axis are tightly regulated. Disruption of HPA axis regulation is known to contribute to a number of stress-related disorders. For example, increased cortisol (hypercortisolism) has been shown in patients with major depressive disorder (MDD) [3,4], and decreased cortisol (hypocortisolism) has been observed in people with post-traumatic stress disorder (PTSD), Gulf War illness, post infection fatigue and chronic fatigue syndrome (CFS) [5-9]. While it is not clear if dysregulation of the HPA axis is a primary or secondary effect of these disorders, there is evidence that stress-related disorders are influenced by early life adverse experiences that affect the neural architecture and gene expression in the brain [10]. Childhood events such as severe infection, malnutrition, physical, sexual and emotional abuse are associated with many chronic illnesses later in life [11].

Definitive research on HPA axis function in chronic diseases has been hampered by the complexity of the numerous systems affected by the HPA axis, such as the immune and neuroendocrine systems, the lack of known or accessible brain lesions and the correlative nature of much of the existing data. Since the organization of the HPA axis has been characterized to detail the feedback and feed forward signalling that regulates HPA axis function [12], it is a system that is amenable to modelling. Models of the HPA axis have been constructed using deterministic coupled ordinary differential equations [13-17]. These models were successful in capturing features such as negative feedback control and diurnal cycling of the HPA axis. Our goal was to understand the dynamic effects of CRH, ACTH and cortisol with a mathematically parsimonious model to gain insight into HPA axis regulation. This model is novel in that it incorporates expression of the glucocorticoid receptor (GR) in the pituitary and demonstrates that repeated stress and GR expression reveals the bistability inherent in the HPA axis given the enhanced model.

### Model

The HPA axis has three compartments representing the hypothalamus, pituitary and adrenals regulated by simple, linear mass action kinetics for the production and degradation of the primary chemical product of each compartment. In this model, stress to the HPA axis (F) stimulates the hypothalamus to secrete CRH (C). CRH (C) signals the induction of ACTH synthesis (A) in the pituitary. ACTH (A) signals to the adrenal gland and activates the synthesis and release of cortisol (O). Cortisol (O) regulates its own synthesis via inhibiting the synthesis of CRH (C) in the hypothalamus, and ACTH (A) in the pituitary. The equation for the hypothalamus can be written as:

dCdT=(Kc+F)∗(1−OKi1)−KcdC     (1)
 MathType@MTEF@5@5@+=feaafiart1ev1aaatCvAUfKttLearuWrP9MDH5MBPbIqV92AaeXatLxBI9gBaebbnrfifHhDYfgasaacH8akY=wiFfYdH8Gipec8Eeeu0xXdbba9frFj0=OqFfea0dXdd9vqai=hGuQ8kuc9pgc9s8qqaq=dirpe0xb9q8qiLsFr0=vr0=vr0dc8meaabaqaciaacaGaaeqabaqabeGadaaakeaadaWcaaqaaiabdsgaKjabdoeadbqaaiabdsgaKjabdsfaubaacqGH9aqpcqGGOaakcqWGlbWsdaWgaaWcbaGaem4yamgabeaakiabgUcaRiabdAeagjabcMcaPiabgEHiQiabcIcaOiabigdaXiabgkHiTmaalaaabaGaem4ta8eabaGaem4saS0aaSbaaSqaaiabdMgaPjabigdaXaqabaaaaOGaeiykaKIaeyOeI0Iaem4saS0aaSbaaSqaaiabdogaJjabdsgaKbqabaGccqWGdbWqcaWLjaGaaCzcamaabmaabaGaeGymaedacaGLOaGaayzkaaaaaa@4BF4@

In this equation, -*K*_*cd*_*C *models a constant degradation rate of CRH in the blood of the portal vein. The term (*K*_*c *_+ *F*)*(1−OKi1)
 MathType@MTEF@5@5@+=feaafiart1ev1aaatCvAUfKttLearuWrP9MDH5MBPbIqV92AaeXatLxBI9gBaebbnrfifHhDYfgasaacH8akY=wiFfYdH8Gipec8Eeeu0xXdbba9frFj0=OqFfea0dXdd9vqai=hGuQ8kuc9pgc9s8qqaq=dirpe0xb9q8qiLsFr0=vr0=vr0dc8meaabaqaciaacaGaaeqabaqabeGadaaakeaacqGGOaakcqaIXaqmcqGHsisldaWcaaqaaiabd+eapbqaaiabdUealnaaBaaaleaacqWGPbqAcqaIXaqmaeqaaaaakiabcMcaPaaa@3512@ models a circadian production term *K*_*c *_and a stress term *F*, both reduced by a linear inhibition term represented by (1−OKi1)
 MathType@MTEF@5@5@+=feaafiart1ev1aaatCvAUfKttLearuWrP9MDH5MBPbIqV92AaeXatLxBI9gBaebbnrfifHhDYfgasaacH8akY=wiFfYdH8Gipec8Eeeu0xXdbba9frFj0=OqFfea0dXdd9vqai=hGuQ8kuc9pgc9s8qqaq=dirpe0xb9q8qiLsFr0=vr0=vr0dc8meaabaqaciaacaGaaeqabaqabeGadaaakeaacqGGOaakcqaIXaqmcqGHsisldaWcaaqaaiabd+eapbqaaiabdUealnaaBaaaleaacqWGPbqAcqaIXaqmaeqaaaaakiabcMcaPaaa@3512@. For small OKi1
 MathType@MTEF@5@5@+=feaafiart1ev1aaatCvAUfKttLearuWrP9MDH5MBPbIqV92AaeXatLxBI9gBaebbnrfifHhDYfgasaacH8akY=wiFfYdH8Gipec8Eeeu0xXdbba9frFj0=OqFfea0dXdd9vqai=hGuQ8kuc9pgc9s8qqaq=dirpe0xb9q8qiLsFr0=vr0=vr0dc8meaabaqaciaacaGaaeqabaqabeGadaaakeaadaWcaaqaaiabd+eapbqaaiabdUealnaaBaaaleaacqWGPbqAcqaIXaqmaeqaaaaaaaa@3179@, we may write (*K*_*c *_+ *F*) * (1−OKi1)
 MathType@MTEF@5@5@+=feaafiart1ev1aaatCvAUfKttLearuWrP9MDH5MBPbIqV92AaeXatLxBI9gBaebbnrfifHhDYfgasaacH8akY=wiFfYdH8Gipec8Eeeu0xXdbba9frFj0=OqFfea0dXdd9vqai=hGuQ8kuc9pgc9s8qqaq=dirpe0xb9q8qiLsFr0=vr0=vr0dc8meaabaqaciaacaGaaeqabaqabeGadaaakeaacqGGOaakcqaIXaqmcqGHsisldaWcaaqaaiabd+eapbqaaiabdUealnaaBaaaleaacqWGPbqAcqaIXaqmaeqaaaaakiabcMcaPaaa@3512@ ≈ Kc+F1+OKi1
 MathType@MTEF@5@5@+=feaafiart1ev1aaatCvAUfKttLearuWrP9MDH5MBPbIqV92AaeXatLxBI9gBaebbnrfifHhDYfgasaacH8akY=wiFfYdH8Gipec8Eeeu0xXdbba9frFj0=OqFfea0dXdd9vqai=hGuQ8kuc9pgc9s8qqaq=dirpe0xb9q8qiLsFr0=vr0=vr0dc8meaabaqaciaacaGaaeqabaqabeGadaaakeaadaWcaaqaaiabdUealnaaBaaaleaacqWGJbWyaeqaaOGaey4kaSIaemOrayeabaGaeGymaeJaey4kaSYaaSaaaeaacqWGpbWtaeaacqWGlbWsdaWgaaWcbaGaemyAaKMaeGymaedabeaaaaaaaaaa@37F6@. The latter form, Kc+F1+OKi1
 MathType@MTEF@5@5@+=feaafiart1ev1aaatCvAUfKttLearuWrP9MDH5MBPbIqV92AaeXatLxBI9gBaebbnrfifHhDYfgasaacH8akY=wiFfYdH8Gipec8Eeeu0xXdbba9frFj0=OqFfea0dXdd9vqai=hGuQ8kuc9pgc9s8qqaq=dirpe0xb9q8qiLsFr0=vr0=vr0dc8meaabaqaciaacaGaaeqabaqabeGadaaakeaadaWcaaqaaiabdUealnaaBaaaleaacqWGJbWyaeqaaOGaey4kaSIaemOrayeabaGaeGymaeJaey4kaSYaaSaaaeaacqWGpbWtaeaacqWGlbWsdaWgaaWcbaGaemyAaKMaeGymaedabeaaaaaaaaaa@37F6@, corresponds to standard linear inhibition of (*K*_*c *_+ *F*) with inhibition constant *K*_*i*1_. This form also guarantees positive ACTH concentrations. We write for the hypothalamus:

dCdT=Kc+F1+OKi1−KcdC     (2)
 MathType@MTEF@5@5@+=feaafiart1ev1aaatCvAUfKttLearuWrP9MDH5MBPbIqV92AaeXatLxBI9gBaebbnrfifHhDYfgasaacH8akY=wiFfYdH8Gipec8Eeeu0xXdbba9frFj0=OqFfea0dXdd9vqai=hGuQ8kuc9pgc9s8qqaq=dirpe0xb9q8qiLsFr0=vr0=vr0dc8meaabaqaciaacaGaaeqabaqabeGadaaakeaadaWcaaqaaiabdsgaKjabdoeadbqaaiabdsgaKjabdsfaubaacqGH9aqpdaWcaaqaaiabdUealnaaBaaaleaacqWGJbWyaeqaaOGaey4kaSIaemOrayeabaGaeGymaeJaey4kaSYaaSaaaeaacqWGpbWtaeaacqWGlbWsdaWgaaWcbaGaemyAaKMaeGymaedabeaaaaaaaOGaeyOeI0Iaem4saS0aaSbaaSqaaiabdogaJjabdsgaKbqabaGccqWGdbWqcaWLjaGaaCzcamaabmaabaGaeGOmaidacaGLOaGaayzkaaaaaa@47A8@

For the pituitary:

dAdT=KaC1+OKi2−KadA     (3)
 MathType@MTEF@5@5@+=feaafiart1ev1aaatCvAUfKttLearuWrP9MDH5MBPbIqV92AaeXatLxBI9gBaebbnrfifHhDYfgasaacH8akY=wiFfYdH8Gipec8Eeeu0xXdbba9frFj0=OqFfea0dXdd9vqai=hGuQ8kuc9pgc9s8qqaq=dirpe0xb9q8qiLsFr0=vr0=vr0dc8meaabaqaciaacaGaaeqabaqabeGadaaakeaadaWcaaqaaiabdsgaKjabdgeabbqaaiabdsgaKjabdsfaubaacqGH9aqpdaWcaaqaaiabdUealnaaBaaaleaacqWGHbqyaeqaaOGaem4qameabaGaeGymaeJaey4kaSYaaSaaaeaacqWGpbWtaeaacqWGlbWsdaWgaaWcbaGaemyAaKMaeGOmaidabeaaaaaaaOGaeyOeI0Iaem4saS0aaSbaaSqaaiabdggaHjabdsgaKbqabaGccqWGbbqqcaWLjaGaaCzcamaabmaabaGaeG4mamdacaGLOaGaayzkaaaaaa@46B4@

Equation 3 models a constant degradation rate of ACTH by the term -*K*_*ad*_*A *and an ACTH production term, KaC1+OKi2
 MathType@MTEF@5@5@+=feaafiart1ev1aaatCvAUfKttLearuWrP9MDH5MBPbIqV92AaeXatLxBI9gBaebbnrfifHhDYfgasaacH8akY=wiFfYdH8Gipec8Eeeu0xXdbba9frFj0=OqFfea0dXdd9vqai=hGuQ8kuc9pgc9s8qqaq=dirpe0xb9q8qiLsFr0=vr0=vr0dc8meaabaqaciaacaGaaeqabaqabeGadaaakeaadaWcaaqaaiabdUealnaaBaaaleaacqWGHbqyaeqaaOGaem4qameabaGaeGymaeJaey4kaSYaaSaaaeaacqWGpbWtaeaacqWGlbWsdaWgaaWcbaGaemyAaKMaeGOmaidabeaaaaaaaaaa@370C@, with a cortisol inhibition factor similar to (2).

For the adrenal:

dOdT=KoA−KodO     (4)
 MathType@MTEF@5@5@+=feaafiart1ev1aaatCvAUfKttLearuWrP9MDH5MBPbIqV92AaeXatLxBI9gBaebbnrfifHhDYfgasaacH8akY=wiFfYdH8Gipec8Eeeu0xXdbba9frFj0=OqFfea0dXdd9vqai=hGuQ8kuc9pgc9s8qqaq=dirpe0xb9q8qiLsFr0=vr0=vr0dc8meaabaqaciaacaGaaeqabaqabeGadaaakeaadaWcaaqaaiabdsgaKjabd+eapbqaaiabdsgaKjabdsfaubaacqGH9aqpcqWGlbWsdaWgaaWcbaGaem4Ba8gabeaakiabdgeabjabgkHiTiabdUealnaaBaaaleaacqWGVbWBcqWGKbazaeqaaOGaem4ta8KaaCzcaiaaxMaadaqadaqaaiabisda0aGaayjkaiaawMcaaaaa@4067@

Equation 4 models a constant degradation rate of cortisol -*K*_*od*_*O *and a cortisol production rate *K*_*o*_*A *linearly dependent on ACTH.

We have augmented this model by including synthesis and regulation of the glucocorticoid receptor (R) in the pituitary [18,19]. In the pituitary, cortisol enters the cell and binds the glucocorticoid receptor in the cytoplasm, causing the receptor to dimerize. This dimerization causes the complex to translocate to the nucleus (dimerization, translocation, and transcription factor binding are not modelled, but assumed to be fast), where it up regulates glucocorticoid receptor (R) synthesis and down regulates production of ACTH (A).

**Figure 1:**
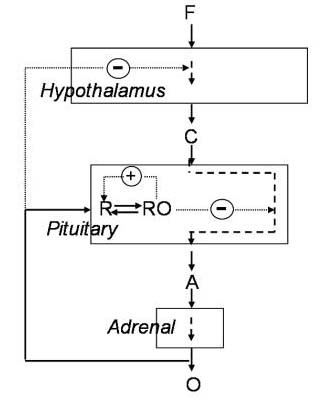
F is an external stress that triggers the hypothalamus to release CRH (C) that signals to the pituitary to release ACTH (A) stimulating the synthesis and release of cortisol (O) from the adrenals. Release of cortisol negatively regulates CRH and ACTH after binding to the glucocorticoid receptor (R) in the pituitary. Here, GR and cortisol regulate further GR synthesis.

The following are the differential equations written for the HPA axis model that includes glucocorticoid receptor synthesis and regulation in the pituitary (Figure 1).

For the hypothalamus:

dCdT=Kc+F1+OKi1−KcdC     (5)
 MathType@MTEF@5@5@+=feaafiart1ev1aaatCvAUfKttLearuWrP9MDH5MBPbIqV92AaeXatLxBI9gBaebbnrfifHhDYfgasaacH8akY=wiFfYdH8Gipec8Eeeu0xXdbba9frFj0=OqFfea0dXdd9vqai=hGuQ8kuc9pgc9s8qqaq=dirpe0xb9q8qiLsFr0=vr0=vr0dc8meaabaqaciaacaGaaeqabaqabeGadaaakeaadaWcaaqaaiabdsgaKjabdoeadbqaaiabdsgaKjabdsfaubaacqGH9aqpdaWcaaqaaiabdUealnaaBaaaleaacqWGJbWyaeqaaOGaey4kaSIaemOrayeabaGaeGymaeJaey4kaSYaaSaaaeaacqWGpbWtaeaacqWGlbWsdaWgaaWcbaGaemyAaKMaeGymaedabeaaaaaaaOGaeyOeI0Iaem4saS0aaSbaaSqaaiabdogaJjabdsgaKbqabaGccqWGdbWqcaWLjaGaaCzcamaabmaabaGaeGynaudacaGLOaGaayzkaaaaaa@47AE@

For the pituitary:

dAdT=KaC1+ORKi2−KadA     (6)
 MathType@MTEF@5@5@+=feaafiart1ev1aaatCvAUfKttLearuWrP9MDH5MBPbIqV92AaeXatLxBI9gBaebbnrfifHhDYfgasaacH8akY=wiFfYdH8Gipec8Eeeu0xXdbba9frFj0=OqFfea0dXdd9vqai=hGuQ8kuc9pgc9s8qqaq=dirpe0xb9q8qiLsFr0=vr0=vr0dc8meaabaqaciaacaGaaeqabaqabeGadaaakeaadaWcaaqaaiabdsgaKjabdgeabbqaaiabdsgaKjabdsfaubaacqGH9aqpdaWcaaqaaiabdUealnaaBaaaleaacqWGHbqyaeqaaOGaem4qameabaGaeGymaeJaey4kaSYaaSaaaeaacqWGpbWtcqWGsbGuaeaacqWGlbWsdaWgaaWcbaGaemyAaKMaeGOmaidabeaaaaaaaOGaeyOeI0Iaem4saS0aaSbaaSqaaiabdggaHjabdsgaKbqabaGccqWGbbqqcaWLjaGaaCzcamaabmaabaGaeGOnaydacaGLOaGaayzkaaaaaa@47E7@

dRdT=Kr(OR)2K+(OR)2+Kcr−KrdR     (7)
 MathType@MTEF@5@5@+=feaafiart1ev1aaatCvAUfKttLearuWrP9MDH5MBPbIqV92AaeXatLxBI9gBaebbnrfifHhDYfgasaacH8akY=wiFfYdH8Gipec8Eeeu0xXdbba9frFj0=OqFfea0dXdd9vqai=hGuQ8kuc9pgc9s8qqaq=dirpe0xb9q8qiLsFr0=vr0=vr0dc8meaabaqaciaacaGaaeqabaqabeGadaaakeaadaWcaaqaaiabdsgaKjabdkfasbqaaiabdsgaKjabdsfaubaacqGH9aqpdaWcaaqaaiabdUealnaaBaaaleaacqWGYbGCaeqaaOGaeiikaGIaem4ta8KaemOuaiLaeiykaKYaaWbaaSqabeaacqaIYaGmaaaakeaacqWGlbWscqGHRaWkcqGGOaakcqWGpbWtcqWGsbGucqGGPaqkdaahaaWcbeqaaiabikdaYaaaaaGccqGHRaWkcqWGlbWsdaWgaaWcbaGaem4yamMaemOCaihabeaakiabgkHiTiabdUealnaaBaaaleaacqWGYbGCcqWGKbazaeqaaOGaemOuaiLaaCzcaiaaxMaadaqadaqaaiabiEda3aGaayjkaiaawMcaaaaa@50DC@

For the adrenal:

dOdT=KoA−KodO     (8)
 MathType@MTEF@5@5@+=feaafiart1ev1aaatCvAUfKttLearuWrP9MDH5MBPbIqV92AaeXatLxBI9gBaebbnrfifHhDYfgasaacH8akY=wiFfYdH8Gipec8Eeeu0xXdbba9frFj0=OqFfea0dXdd9vqai=hGuQ8kuc9pgc9s8qqaq=dirpe0xb9q8qiLsFr0=vr0=vr0dc8meaabaqaciaacaGaaeqabaqabeGadaaakeaadaWcaaqaaiabdsgaKjabd+eapbqaaiabdsgaKjabdsfaubaacqGH9aqpcqWGlbWsdaWgaaWcbaGaem4Ba8gabeaakiabdgeabjabgkHiTiabdUealnaaBaaaleaacqWGVbWBcqWGKbazaeqaaOGaem4ta8KaaCzcaiaaxMaadaqadaqaaiabiIda4aGaayjkaiaawMcaaaaa@406F@

Equation (7) describes the production of GR in the pituitary. The term Kr(OR)2K+(OR)2
 MathType@MTEF@5@5@+=feaafiart1ev1aaatCvAUfKttLearuWrP9MDH5MBPbIqV92AaeXatLxBI9gBaebbnrfifHhDYfgasaacH8akY=wiFfYdH8Gipec8Eeeu0xXdbba9frFj0=OqFfea0dXdd9vqai=hGuQ8kuc9pgc9s8qqaq=dirpe0xb9q8qiLsFr0=vr0=vr0dc8meaabaqaciaacaGaaeqabaqabeGadaaakeaadaWcaaqaaiabdUealnaaBaaaleaacqWGYbGCaeqaaOGaeiikaGIaem4ta8KaemOuaiLaeiykaKYaaWbaaSqabeaacqaIYaGmaaaakeaacqWGlbWscqGHRaWkcqGGOaakcqWGpbWtcqWGsbGucqGGPaqkdaahaaWcbeqaaiabikdaYaaaaaaaaa@3BD3@ in equation 7 is in Michaelis-Menten form since we assume the bound glucocorticoid receptor (OR) dimerizes with fast kinetics, so that the amount of dimer is in constant quasi-equilibrium, depending on the abundance of OR and the equilibrium binding affinity (*K*). The model further assumes that cortisol (O) and the glucocorticoid receptor (R) bind to each other with very fast kinetics compared to the rate of change of the 4 state variables (A, C, O, and R), so that OR stays in quasi-equilibrium as well. These are reasonable assumptions, given that high affinity receptor-ligand kinetics are often much faster than enzyme kinetics (as is assumed in the standard Michaelis-Menten equation) or than steps requiring transcription and/or translation for protein synthesis. Equation (7) also models a linear production term *K*_*cr *_and a degradation term -*K*_*rd*_*R *for pituitary GR production. Equation (6) reflects the inhibition dependence of glucocorticoid receptor (R) and cortisol (O) with an inhibition constant *K*_*i*2_.

Scaling of the equations (5) – (8) has been done to reduce the parameters used in simulations. The scaled variables are defined as;

t=KodT,c=KodCKc,a=Kod2AKcKa,o=Kod3OKcKaKo
 MathType@MTEF@5@5@+=feaafiart1ev1aaatCvAUfKttLearuWrP9MDH5MBPbIqV92AaeXatLxBI9gBaebbnrfifHhDYfgasaacH8akY=wiFfYdH8Gipec8Eeeu0xXdbba9frFj0=OqFfea0dXdd9vqai=hGuQ8kuc9pgc9s8qqaq=dirpe0xb9q8qiLsFr0=vr0=vr0dc8meaabaqaciaacaGaaeqabaqabeGadaaakeaacqWG0baDcqGH9aqpcqWGlbWsdaWgaaWcbaGaem4Ba8MaemizaqgabeaakiabdsfaujabcYcaSiabdogaJjabg2da9maalaaabaGaem4saS0aaSbaaSqaaiabd+gaVjabdsgaKbqabaGccqWGdbWqaeaacqWGlbWsdaWgaaWcbaGaem4yamgabeaaaaGccqGGSaalcqWGHbqycqGH9aqpdaWcaaqaaiabdUealnaaDaaaleaacqWGVbWBcqWGKbazaeaacqaIYaGmaaGccqWGbbqqaeaacqWGlbWsdaWgaaWcbaGaem4yamgabeaakiabdUealnaaBaaaleaacqWGHbqyaeqaaaaakiabcYcaSiabd+gaVjabg2da9maalaaabaGaem4saS0aa0baaSqaaiabd+gaVjabdsgaKbqaaiabiodaZaaakiabd+eapbqaaiabdUealnaaBaaaleaacqWGJbWyaeqaaOGaem4saS0aaSbaaSqaaiabdggaHbqabaGccqWGlbWsdaWgaaWcbaGaem4Ba8gabeaaaaaaaa@5F72@

r=KodRKr,kcd=KcdKod,kad=KadKod,krd=KrdKod
 MathType@MTEF@5@5@+=feaafiart1ev1aaatCvAUfKttLearuWrP9MDH5MBPbIqV92AaeXatLxBI9gBaebbnrfifHhDYfgasaacH8akY=wiFfYdH8Gipec8Eeeu0xXdbba9frFj0=OqFfea0dXdd9vqai=hGuQ8kuc9pgc9s8qqaq=dirpe0xb9q8qiLsFr0=vr0=vr0dc8meaabaqaciaacaGaaeqabaqabeGadaaakeaacqWGYbGCcqGH9aqpdaWcaaqaaiabdUealnaaBaaaleaacqWGVbWBcqWGKbazaeqaaOGaemOuaifabaGaem4saS0aaSbaaSqaaiabdkhaYbqabaaaaOGaeiilaWIaem4AaS2aaSbaaSqaaiabdogaJjabdsgaKbqabaGccqGH9aqpdaWcaaqaaiabdUealnaaBaaaleaacqWGJbWycqWGKbazaeqaaaGcbaGaem4saS0aaSbaaSqaaiabd+gaVjabdsgaKbqabaaaaOGaeiilaWIaem4AaS2aaSbaaSqaaiabdggaHjabdsgaKbqabaGccqGH9aqpdaWcaaqaaiabdUealnaaBaaaleaacqWGHbqycqWGKbazaeqaaaGcbaGaem4saS0aaSbaaSqaaiabd+gaVjabdsgaKbqabaaaaOGaeiilaWIaem4AaS2aaSbaaSqaaiabdkhaYjabdsgaKbqabaGccqGH9aqpdaWcaaqaaiabdUealnaaBaaaleaacqWGYbGCcqWGKbazaeqaaaGcbaGaem4saS0aaSbaaSqaaiabd+gaVjabdsgaKbqabaaaaaaa@61DC@

The scaled equations thereby obtained are;

dcdt=1+f1+oki1−kcdc     (9)
 MathType@MTEF@5@5@+=feaafiart1ev1aaatCvAUfKttLearuWrP9MDH5MBPbIqV92AaeXatLxBI9gBaebbnrfifHhDYfgasaacH8akY=wiFfYdH8Gipec8Eeeu0xXdbba9frFj0=OqFfea0dXdd9vqai=hGuQ8kuc9pgc9s8qqaq=dirpe0xb9q8qiLsFr0=vr0=vr0dc8meaabaqaciaacaGaaeqabaqabeGadaaakeaadaWcaaqaaiabdsgaKjabdogaJbqaaiabdsgaKjabdsha0baacqGH9aqpdaWcaaqaaiabigdaXiabgUcaRiabdAgaMbqaaiabigdaXiabgUcaRmaalaaabaGaem4Ba8gabaGaem4AaS2aaSbaaSqaaiabdMgaPjabigdaXaqabaaaaaaakiabgkHiTiabdUgaRnaaBaaaleaacqWGJbWycqWGKbazaeqaaOGaem4yamMaaCzcaiaaxMaadaqadaqaaiabiMda5aGaayjkaiaawMcaaaaa@47C2@

dadt=c1+orki2−kada     (10)
 MathType@MTEF@5@5@+=feaafiart1ev1aaatCvAUfKttLearuWrP9MDH5MBPbIqV92AaeXatLxBI9gBaebbnrfifHhDYfgasaacH8akY=wiFfYdH8Gipec8Eeeu0xXdbba9frFj0=OqFfea0dXdd9vqai=hGuQ8kuc9pgc9s8qqaq=dirpe0xb9q8qiLsFr0=vr0=vr0dc8meaabaqaciaacaGaaeqabaqabeGadaaakeaadaWcaaqaaiabdsgaKjabdggaHbqaaiabdsgaKjabdsha0baacqGH9aqpdaWcaaqaaiabdogaJbqaaiabigdaXiabgUcaRmaalaaabaGaem4Ba8MaemOCaihabaGaem4AaS2aaSbaaSqaaiabdMgaPjabikdaYaqabaaaaaaakiabgkHiTiabdUgaRnaaBaaaleaacqWGHbqycqWGKbazaeqaaOGaemyyaeMaaCzcaiaaxMaadaqadaqaaiabigdaXiabicdaWaGaayjkaiaawMcaaaaa@482B@

drdt=(or)2k+(or)2+kcr−krdr     (11)
 MathType@MTEF@5@5@+=feaafiart1ev1aaatCvAUfKttLearuWrP9MDH5MBPbIqV92AaeXatLxBI9gBaebbnrfifHhDYfgasaacH8akY=wiFfYdH8Gipec8Eeeu0xXdbba9frFj0=OqFfea0dXdd9vqai=hGuQ8kuc9pgc9s8qqaq=dirpe0xb9q8qiLsFr0=vr0=vr0dc8meaabaqaciaacaGaaeqabaqabeGadaaakeaadaWcaaqaaiabdsgaKjabdkhaYbqaaiabdsgaKjabdsha0baacqGH9aqpdaWcaaqaaiabcIcaOiabd+gaVjabdkhaYjabcMcaPmaaCaaaleqabaGaeGOmaidaaaGcbaGaem4AaSMaey4kaSIaeiikaGIaem4Ba8MaemOCaiNaeiykaKYaaWbaaSqabeaacqaIYaGmaaaaaOGaey4kaSIaem4AaS2aaSbaaSqaaiabdogaJjabdkhaYbqabaGccqGHsislcqWGRbWAdaWgaaWcbaGaemOCaiNaemizaqgabeaakiabdkhaYjaaxMaacaWLjaWaaeWaaeaacqaIXaqmcqaIXaqmaiaawIcacaGLPaaaaaa@517E@

dodt=a−o     (12)
 MathType@MTEF@5@5@+=feaafiart1ev1aaatCvAUfKttLearuWrP9MDH5MBPbIqV92AaeXatLxBI9gBaebbnrfifHhDYfgasaacH8akY=wiFfYdH8Gipec8Eeeu0xXdbba9frFj0=OqFfea0dXdd9vqai=hGuQ8kuc9pgc9s8qqaq=dirpe0xb9q8qiLsFr0=vr0=vr0dc8meaabaqaciaacaGaaeqabaqabeGadaaakeaadaWcaaqaaiabdsgaKjabd+gaVbqaaiabdsgaKjabdsha0baacqGH9aqpcqWGHbqycqGHsislcqWGVbWBcaWLjaGaaCzcamaabmaabaGaeGymaeJaeGOmaidacaGLOaGaayzkaaaaaa@3B8A@

These scaled equations were used in the simulations. The advantage of scaling is that it obviates the need for knowledge of unknown parameter values such as the synthesis rate of CRH in the hypothalamus and ACTH and GR in the pituitary. The parameter values that can be measured are the degradation rates of CRH, ACTH, and cortisol. The scaled parameter values used in simulation were, *k*_*cd *_= 1, *k*_*ad *_= 10, *k*_*rd *_= 0.9, *k*_*cr *_= 0.05, *k *= 0.001, *k*_*i*1 _= 0.1, and *k*_*i*2 _= 0.1. Further, these simulated results for CRH, ACTH and cortisol are converted back to their commonly used dimensions and values obtained in experiments. The simulated time course plots ignore the circadian input to the hypothalamus.

Models were programmed in Matlab (The Mathworks, Natick, MA). The meta-modeling of bi-stability used the CONTENT freeware package. All Matlab code will be provided upon request. Dr. Leslie Crofford provided the human subject serum cortisol data [9].

## Results

**Figure 2:**
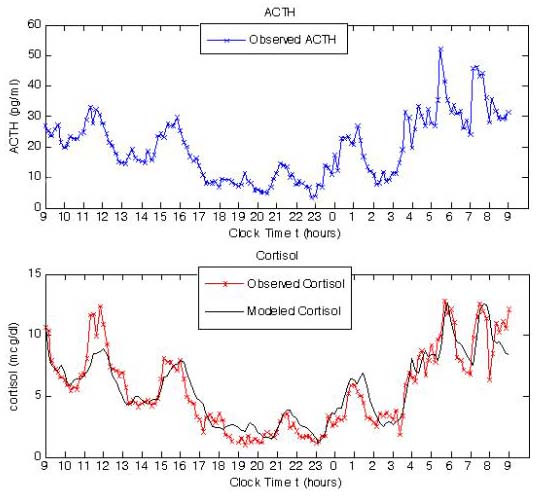
Experimental ACTH and cortisol from a human subject shown in blue and red in top and bottom panels respectively. Modelled cortisol using equation 4 displayed with solid black line in lower panel.

To determine if these equations could predict the general features of cortisol production, the experimental data was compared to a cortisol curve generated using equation 4. As shown in Figure 2, equation 4 predicts a fit that is very similar to the actual cortisol production in this healthy human subject. Experimental fitting of ACTH is not possible since hypothalamic derived CRH cannot be measured.

### Steady States

**Figure 3:**
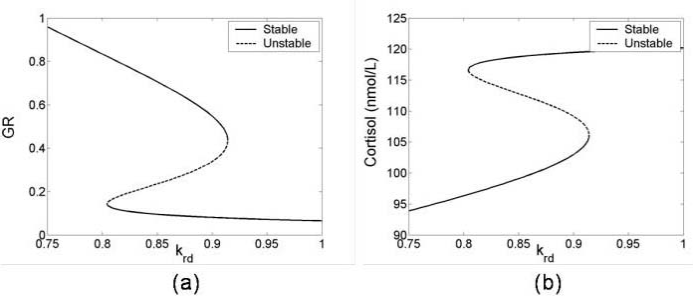
Variations of steady state (a) GR and (b) cortisol with *k*_*rd*_. Solid and dashed lines denote the stable and unstable steady states, respectively. If *k*_*rd *_for a given patient is in the region where GR and cortisol are multivalued, then the given patient can be pushed from one value of steady state GR or cortisol to equally valid altered steady state levels by the application of an extreme stress.

Equations (9)–(12) permit one or three positive steady states depending upon the parameter values. The three positive steady states exist because of homodimerization of the GR with cortisol. Figure 3 shows the variation of GR and cortisol steady state with respect to parameter *k*_*rd*_. Variations in *k*_*rd *_from person to person may be expected due to genetic differences in the details of GR production and degradation. For a high value of *k*_*rd*_, there exists only a low GR concentration steady state. As the value of *k*_*rd *_decreases, these equations produce two more steady states, one stable and another unstable in GR concentration. As *k*_*rd *_decreases further, a low GR concentration state disappears and only a high GR concentration state exists (Figure 3a). In this model, we postulate that the low GR concentration represents the normal steady state, and high GR concentration denotes a dysregulated HPA axis steady state as it results in persistent low cortisol levels (hypocortisolism) (Figure 3b). Hypocortisolism results from the negative feedback between GR (i.e. the symbol "R" in Figure 1) and ACTH (A), and hence cortisol (O) produced downstream of it, as shown in Figure 1 and reflected by the inverse relationship between cortisol and GR in Figure 3. Thus individuals with very large values of *k*_*rd *_would be constitutively healthy in this model, i.e. impervious to a dysregulated HPA-axis no matter how much they are stressed, and those with very low values of *k*_*rd *_would be constitutively unhealthy.

### Normal stress response

**Figure 4:**
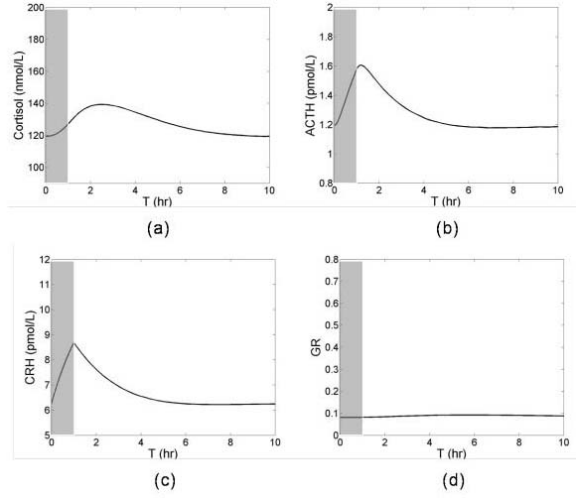
The response of the HPA axis following a short stress. Short time stress as indicated by the shaded larea was given for 0<T<1 hr.

The response of the normal HPA axis to small perturbations is essential to the survival of an organism. Stress activates the HPA axis to regulate various body functions; first by increasing ACTH synthesis followed by increased cortisol production and then returning to the original state. Figure 4 shows a simulation of the response of the HPA axis to a short stress. The initial condition of the HPA axis was set to a normal steady state and at T = 0, a stress was given for 0<T<1. The HPA axis responded to this disturbance by secreting CRH. The synthesis of CRH induced the synthesis of ACTH and cortisol (Figures 4a and 4b). The synthesis of CRH stopped once the stress ended, and the concentration of CRH quickly decreased due to CRH degradation (Figure 4c). CRH returned to steady state meanwhile stimulating the release of ACTH that also peaked shortly after the short stress ended (Figure 4b). Synthesis of cortisol followed the peak ACTH secretion (Figure 4a). The concentration of GR was only slightly elevated following the short stress and then returned to baseline (Figure 4d).

### Adaptation of HPA axis

**Figure 5:**
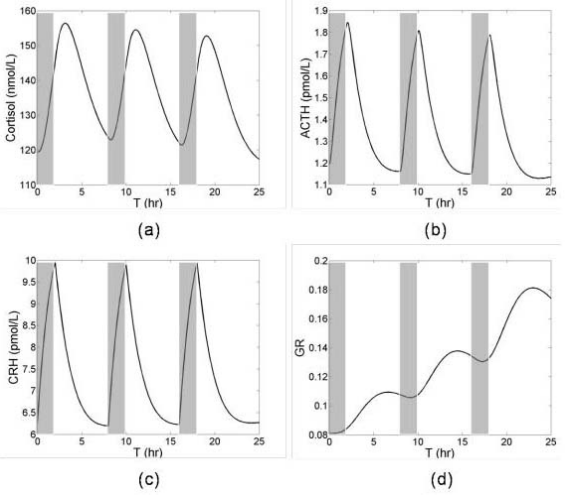
Transient responses of HPA axis to recursive stresses. Initially HPA axis was at a lower GR steady state and stress was given at T = 0, 8 and 16 for 2 hours. Repeated stresses are shown by shaded areas.

The robustness of the system was illustrated by the fact that short stress produced small transients that returned to the original, normal steady state. To simulate adaptation of the HPA axis to repeated stress, recursive stress was applied at T = 0, 8 and 16 hours for 2 hour periods. The simulation results showed the continuous decrease in maximum ACTH and cortisol concentration after every stress (Figure 5a and 5b) while CRH is relatively unaffected (Figure 5c). The decrease in secretion of ACTH and cortisol occurred because of an increase in pituitary GR concentration and the fact that the system was pulsed with the stresses before it had time to fully recover (Figure 5d).

### Chronic stress response

**Figure 6:**
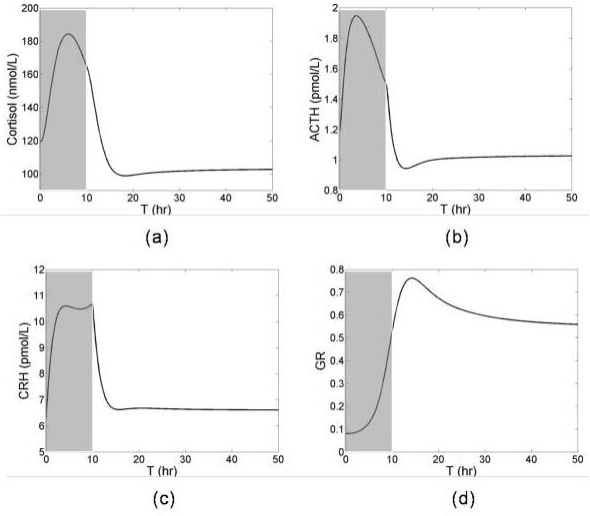
Transient responses of HPA axis to chronic stress. Extended length stress was given for 0<T<10. Stress is indicated with shading.

To simulate the response to chronic stress, a long stress was given for 0<T<10 hours to perturb the normal steady state of the HPA axis. Simulation results show the bistability in the HPA axis; a long stress forces the HPA axis to an alternate steady state (Figure 6). The HPA axis secreted cortisol in response to stress. The increased concentration of cortisol induced the synthesis of GR and the inhibition of pituitary ACTH. When stress was applied for long periods, GR synthesis continued and crossed the threshold middle unstable steady state of GR (Figure 3a). At this point, the HPA axis reached the basin of attraction of the second stable steady state and remained there even after the removal of stress. The higher concentration of GR triggered further pituitary ACTH inhibition, resulting in a lower basal level ACTH and cortisol production (Figures 6a and 6b).

### HPA axis challenge

**Figure 7:**
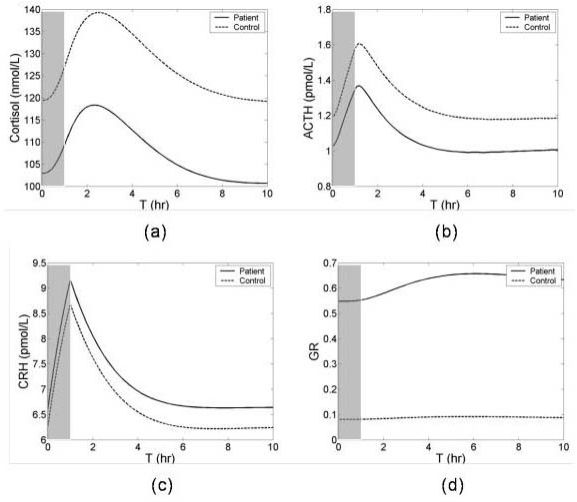
Transient responses of HPA axis a simulated stress experiment. The same stress was given with two different initial conditions; normal steady state (low GR concentration) that would occur in a control group, and low cortisol state (high GR concentration) that would occur in a patient group. Stress was given for 0<Time<1 hr. Dash and solid lines indicate the normal and dysregulated HPA axis responses respectively and stress is indicated with shading.

**Figure 8:**
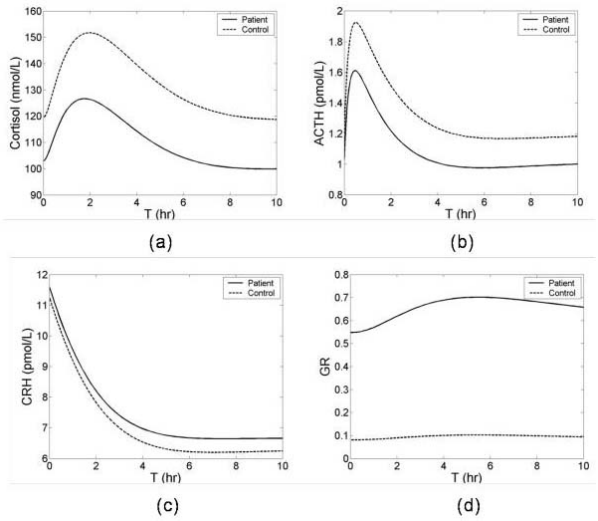
Transient responses of HPA axis to CRH test. The exogenous CRH was injected at T = 0. Dashed and solid lines indicate the normal and dysregulated HPA axis responses respectively.

Psychologic stress, CRH and dexamethasone (DEX) tests are used to assess HPA axis function. The model was used to simulate these various HPA axis function tests. To simulate a psychologic stress experiment, the same stress was given with two different initial conditions: normal steady state (low GR concentration) that would occur in a control group, and low cortisol state (high GR concentration) that would occur in a hypocortisolemic patient group. Because the high concentration GR inhibited ACTH synthesis, the patient group exhibited continued low cortisol and ACTH responses compared to the control (Figures 7a and 7b). To simulate the CRH test, e.g., one that requires exogenous CRH administration, CRH concentration was increased by a constant amount. This resulted in increased pituitary and adrenal gland synthesis of ACTH and cortisol respectively. The high concentration of pituitary GR in the patient group blunted both responses compared to the control (Figures 8a and 8b) Both Figures 7 and 8 demonstrate that the model behaves in a qualitatively similar fashion to observed experimental results.

## Discussion

Previous models of the HPA axis have not demonstrated bistability in steady state cortisol or ACTH. We believe this is because none of the previous models have explicitly accounted for nonlinear kinetics, such as the homodimerization of GR after cortisol activation [18,19]. This is essential for the negative feedback control of the HPA axis. This homodimerization engenders the existence of two stable steady states and one unstable steady state in GR expression in the pituitary. While increased cortisol following a short period of stress produces a small perturbation in GR concentration, long and repeated periods of stress resulting in elevated cortisol levels produce a large perturbation in GR concentration that force the HPA axis into an alternate steady state. Because of the existence of two stable steady states in this model, a small increase GR concentration can be regulated, but a large perturbation in GR concentration is sustained even after the removal of the long duration stress. A higher concentration of GR increases the concentration of cortisol-GR complexes that in turn enhance the inhibition of ACTH synthesis in the pituitary. Since ACTH stimulates the production of cortisol, less ACTH results in lower cortisol secretion and a decrease HPA axis activity.

GR is found in cells throughout the human brain and body. However, GR synthesis and regulation is tissue and organ specific. For example, while corticosterone injection in rats inhibits the synthesis of GR-mRNA in lymphocyte, hypothalamic and hippocampal cells [20,21], it induces the synthesis of GR-mRNA and increases the sensitivity in the anterior pituitary [22,23]. Our model incorporates the increased synthesis of GR in the anterior pituitary. Increased GR makes anterior pituitary cells more sensitive to cortisol and enhances the negative feedback effect of cortisol on ACTH production. Enhanced negative feedback control of ACTH production in the anterior pituitary may produce a hypocortisol state.

We were also able to demonstrate that these simulation results are qualitatively similar to cortisol levels measured in a human subject (Figure 2). A large number of studies have investigated alterations of the HPA axis in CFS, including both studies of basal HPA axis activity as well as studies of HPA axis responsiveness to challenge (for review see [24]). A hypocortisol steady state, such as was demonstrated in this modelling and simulation study, is in keeping with many of these studies

There may be other physiologically plausible mechanisms that produce bi-stability other than the anterior pituitary GR homodimerization mechanism investigated here. The point of this investigation is not to conclusively prove that pituitary GR dimerization is the cause of hypocortisolism, but rather to demonstrate that there are physiologically plausible mechanisms for producing bistability in the HPA-axis that are stress modulated. Further mining of the experimental literature together with mathematical modelling will reveal additional plausible mechanisms.

## Conclusion

Moderate, short-lived stress responses that result in transient increases in cortisol are important and necessary for maintaining body homeostasis and health. Strong and prolonged stress can force the HPA axis into an altered steady state. We demonstrate bistability in the HPA axis due to pituitary GR synthesis. This altered steady state, characterized by hypocortisolism, is observed in a number of stress-related illnesses. The elucidation of bistability in this model of the HPA axis through the action of pituitary GR effects may lead to targeted treatments of stress-related illness where hypocortisolism is the primary clinical manifestation.

## Authors' contributions

SG was responsible for programming the differential equation models, producing the mathematics for the meta-analysis on stress response and bistability, and writing of the manuscript. EA and SDV were responsible for the concept, the design of this study and preparation, validation, writing, and critical review of the manuscript. BMG provided assistance on the mathematical analysis and was responsible for critical review and editing of the manuscript.

## Disclaimer

The findings and conclusions in this report are those of the author(s) and do not necessarily represent the views of the funding agency.

## Declaration of competing interests

The author(s) declare that they have no competing interests.

## References

[B1] Munck A, Guyre PM, Holbrook NJ (1984). Physiological functions of glucocorticoids in stress and their relation to pharmacological actions. Endocr Rev.

[B2] Tuckermann JP, Kleiman A, McPherson KG, Reichardt HM (2005). Molecular mechanisms of glucocorticoids in the control of inflammation and lymphocyte apoptosis. Crit Rev Clin Lab Sci.

[B3] Juruena MF, Cleare AJ, Pariante CM (2004). The hypothalamic pituitary adrenal axis, glucocorticoid receptor function and relevance to depression. Rev Bras Psiquiatr.

[B4] Gold PW, Chrousos GP (2002). Organization of the stress system and its dysregulation in melancholic and atypical depression: high vs low CRH/NE states. Mol Psychiatry.

[B5] Rohleder N, Joksimovic L, Wolf JM, Kirschbaum C (2004). Hypocortisolism and increased glucocorticoid sensitivity of pro-inflammatory cytokine production in Bosnian war refugees with posttraumatic stress disorder. Biol Psychiatry.

[B6] Demitrack MA, Dale JK, Straus SE, Laue L, Listwak SJ, Kruesi MJ (1991). Evidence for impaired activation of the hypothalamic-pituitary-adrenal axis in patients with chronic fatigue syndrome. J Clin Endocrinol Metab.

[B7] Di GA, Hudson M, Jerjes W, Cleare AJ (2005). 24-hour pituitary and adrenal hormone profiles in chronic fatigue syndrome. Psychosom Med.

[B8] Jerjes WK, Peters TJ, Taylor NF, Wood PJ, Wessely S, Cleare AJ (2006). Diurnal excretion of urinary cortisol, cortisone, and cortisol metabolites in chronic fatigue syndrome. J Psychosom Res.

[B9] Crofford LJ, Young EA, Engleberg NC, Korszun A, Brucksch CB, McClure LA, Brown MB, Demitrack MA (2004). Basal circadian and pulsatile ACTH and cortisol secretion in patients with fibromyalgia and/or chronic fatigue syndrome. Brain Behav Immun.

[B10] (2006). National Scientific Council on the Developing Child, Early Exposure to Toxic Substances Damages Brain Architecture. Working Paper No 4.

[B11] Turner-Cobb JM (2005). Psychological and stress hormone correlates in early life: a key to HPA-axis dysregulation and normalisation. Stress.

[B12] Jacobson L (2005). Hypothalamic-pituitary-adrenocortical axis regulation. Endocrinol Metab Clin North Am.

[B13] Gonzalez-Heydrich J, Steingard RJ, Kohane I (1994). A computer simulation of the hypothalamic-pituitary-adrenal axis. Proc Annu Symp Comput Appl Med Care.

[B14] Dempsher DP, Gann DS, Phair RD (1984). A mechanistic model of ACTH-stimulated cortisol secretion. Am J Physiol.

[B15] Sharma DC, Gabrilove JL (1975). A study of the adrenocortical disorders related to the biosynthesis and regulation of steroid hormones and their computer simulation. Mt Sinai J Med.

[B16] Savic D (2005). A mathematical model of the hypothalamo-pituitary-adrenocortical system and its stability analysis. Chaos, solitons, and fractals.

[B17] Lenbury Y, Pornsawad P (2005). A delay-differential equation model of the feedback-controlled hypothalamus-pituitary-adrenal axis in humans. Math Med Biol.

[B18] Drouin J, Sun YL, Tremblay S, Lavender P, Schmidt TJ, de LA (1992). Homodimer formation is rate-limiting for high affinity DNA binding by glucocorticoid receptor. Mol Endocrinol.

[B19] Tsai SY, Carlstedt-Duke J, Weigel NL, Dahlman K, Gustafsson JA, Tsai MJ (1988). Molecular interactions of steroid hormone receptor with its enhancer element: evidence for receptor dimer formation. Cell.

[B20] Makino S, Smith MA, Gold PW (1995). Increased expression of corticotropin-releasing hormone and vasopressin messenger ribonucleic acid (mRNA) in the hypothalamic paraventricular nucleus during repeated stress: association with reduction in glucocorticoid receptor mRNA levels. Endocrinology.

[B21] Nishimura K, Makino S, Tanaka Y, Kaneda T, Hashimoto K (2004). Altered expression of p53 mRNA in the brain and pituitary during repeated immobilization stress: negative correlation with glucocorticoid receptor mRNA levels. J Neuroendocrinol.

[B22] Hugin-Flores ME, Steimer T, Aubert ML, Schulz P (2004). Mineralo- and glucocorticoid receptor mRNAs are differently regulated by corticosterone in the rat hippocampus and anterior pituitary. Neuroendocrinology.

[B23] Dayanithi G, Antoni FA (1989). Rapid as well as delayed inhibitory effects of glucocorticoid hormones on pituitary adrenocorticotropic hormone release are mediated by type II glucocorticoid receptors and require newly synthesized messenger ribonucleic acid as well as protein. Endocrinology.

[B24] Cleare AJ (2004). The HPA axis and the genesis of chronic fatigue syndrome. Trends Endocrinol Metab.

